# Antibody–Drug Conjugates Using Mouse–Canine Chimeric Anti-Dog Podoplanin Antibody Exerts Antitumor Activity in a Mouse Xenograft Model

**DOI:** 10.1089/mab.2020.0001

**Published:** 2020-04-09

**Authors:** Yukinari Kato, Yuji Ito, Tomokazu Ohishi, Manabu Kawada, Takuro Nakamura, Yusuke Sayama, Masato Sano, Teizo Asano, Miyuki Yanaka, Saki Okamoto, Saori Handa, Yu Komatsu, Junko Takei, Mika K. Kaneko

**Affiliations:** ^1^ Department of Antibody Drug Development, Tohoku University Graduate School of Medicine, Sendai, Japan.; ^2^ New Industry Creation Hatchery Center, Tohoku University, Sendai, Japan.; ^3^ Department of Chemistry and Bioscience, Graduate School of Science and Engineering, Kagoshima University, Kagoshima, Japan.; ^4^ Institute of Microbial Chemistry (BIKAKEN), Numazu, Microbial Chemistry Research Foundation, Shizuoka, Japan.

**Keywords:** antibody–drug conjugate, dog podoplanin, monoclonal antibody

## Abstract

Antibody–drug conjugates (ADCs), which consist of a monoclonal antibody (mAb), a linker, and a payload, can deliver a drug to cancer tissues. We previously produced an anti-dog podoplanin (dPDPN) mAb, PMab-38, which reacts with dPDPN-expressing canine melanomas and squamous cell carcinomas (SCCs), but not with dPDPN-expressing canine type I alveolar cells or lymphatic endothelial cells, indicating that PMab-38 possesses cancer specificity. In this study, we developed an ADC, P38B-DM1, using the mouse–canine chimeric anti-dPDPN antibody, P38B as the antibody, a peptide linker, and emtansine as the payload using the chemical conjugation by affinity peptide (CCAP) method. We investigated its cytotoxicity against dPDPN-overexpressed Chinese hamster ovary (CHO/dPDPN) cells *in vitro* and its antitumor activity using a mouse xenograft model of CHO/dPDPN cells. P38B-DM1 showed cytotoxicity to CHO/dPDPN cells in a dose-dependent manner *in vitro*. Furthermore, P38B-DM1 exhibited higher antitumor activity than P38B in the mouse xenograft model. These results suggest that P38B-DM1, developed using the CCAP method, is useful for antibody therapy against dPDPN-expressing canine SCCs and melanomas.

## Introduction

Antibody–drug conjugates (ADCs), which consist of an antibody, a linker, and a payload, possess the ability to deliver a drug to specific cells. Several site-specific chemical conjugation strategies with native monoclonal antibodies (mAbs) have been developed, including residue-selective labeling, disulfide rebridging, and affinity-peptide-mediated site-specific chemical conjugation technologies.^(1)^ The most popular antibody conjugation system is an amine coupling, using the activated carboxylate moiety, which has been used to generate a typical ADC, T-DM1 (Kadcyla), used against breast cancers. However, to overcome difficulties involved in controlling the modification site and the drug concentration (drug-to-antibody ratio [DAR]), site-specific modification of the antibody has been favored.^(2)^ Recently, chemical conjugation by affinity peptide (CCAP) has been developed using affinity peptide to the antibody Fc fragment as a novel site-specific conjugation system.^(3)^ Utilizing CCAP, various kinds of therapeutic molecules, from low molecular weight compounds to proteins, can be attached to the antibody Fc through the affinity peptide. In this study, we investigated an ADC consisting of a conjugate between antibody and human serum albumin (HSA)-modified with emtansine (DM1; a tubulin polymerization inhibitor).

Podoplanin (PDPN), which is also known as Aggrus, T1alpha, PA2.26, OTS-8, and gp36, has been reported to be expressed in normal tissues, including podocytes of the kidney, lymphatic endothelial cells, and type I alveolar cells of the lung.^(4)^ PDPN is also overexpressed in many cancers, such as glioblastomas,^(5)^ lung cancers,^(6)^ and mesotheliomas.^(7,8)^ Several clinical studies have investigated the association between PDPN overexpression and poor disease prognosis,^(9)^ suggesting that anti-PDPN mAbs could be used for novel therapeutic strategies against cancer development and metastatic progression.^(10)^

Previously, we established an anti-dog podoplanin (dPDPN), PMab-38 mAb.^(11)^ PMab-38 did not react with lymphatic endothelial cells, and recognized dPDPN of renal epithelial cells weakly^(11)^ although dPDPN is expressed in those cells.^(12)^ Immunohistochemical analyses demonstrated that PMab-38 stained canine squamous cell carcinomas^(13)^ and canine melanomas,^(14)^ indicating that PMab-38 demonstrated cancer specificity. Epitope mapping studies revealed Tyr67 and Glu68 of dPDPN to be the critical epitopes of PMab-38.^(15)^ Furthermore, we produced a mouse–canine chimeric anti-dPDPN antibody, P38B, derived from PMab-38.^(16)^ P38B possesses antibody-dependent cellular cytotoxicity (ADCC) and complement-dependent cytotoxicity (CDC) activities against Chinese hamster ovary (CHO)/dPDPN cells. Although P38B exhibited antitumor activity in mouse xenograft models using CHO/dPDPN,^(17)^ that activity was insufficient for targeting canine cancers. In this study, we investigated whether an ADC of P38B (P38B-DM1) showed superior antitumor activity in a CHO/dPDPN xenograft model.

## Materials and Methods

### Antibodies

PMab-38, a mouse anti-dPDPN mAb was developed in our previous study.^(11)^ To develop the mouse–canine chimeric antibody P38B, V_H_ of PMab-38 and C_H_ of canine immunoglobulin G (IgG) subclass B were subcloned into the pCAG-Ble vector (FUJIFILM Wako Pure Chemical Corporation, Osaka, Japan), and V_L_ of PMab-38 and C_L_ of canine IgG were subcloned into the pCAG-Neo vector (FUJIFILM Wako Pure Chemical Corporation).^(16)^ Using the ExpiFectamine CHO Transfection kit (Thermo Fisher Scientific, Inc., Waltham, MA), the expression vectors were transfected into ExpiCHO-S cells to express P38B antibody. P38B was purified using Protein G-Sepharose (GE Healthcare Biosciences, Pittsburgh, PA).

### Preparation of ADC P38B-DM1

For the preparation of the ADC using P38B, azide-modified CCAP peptide (FNMQQQ(Lys[Azide])RFYEALHDPNLNEEQRNARI(Lys[Azide])SIRDDPSRR(Lys[Azide])RR(Lys[Azide])RR(Lys[Azide])-CONH2), where Lys[Azide] is 5-azide-2-amino pentanoic acid) derived from Z34C peptide^(18)^ was used. This CCAP peptide was N-terminally modified with disuccinimidyl glutarate to give the CCAP reagent (*N*-terminal succinimidyl glutaryl peptide). P38B (4.5 mL, 5.1 mg/mL; 34 μM) in phosphate-buffered saline (PBS) was mixed with 22 μL of 27.9 mM CCAP reagent dissolved in dimethyl sulfoxide and incubated for 1 hour at room temperature.

The reaction of antibody with CCAP peptide generated the monovalent and divalent antibodies owing to the two modified sites of CCAP peptide on Fc. To remove the unmodified and the divalent modified antibodies, the reaction mixture was subjected to affinity chromatography conjugated with Z33 affinity peptide (Acetyl-FNMQQQRRFYEALHDPNLNEEQRNARIRSIRDDPSGSGSK-NH2). The monovalent modified antibody was recovered by eluting with mild acidic buffer. Based on ion-exchange chromatography, the purity of monovalent modified antibody was estimated to be 95%, leaving the residual contaminant of 5% unmodified antibody.

The modification of HSA with DM-1 was performed as follows. HSA (20 mg) dissolved in 1.35 mL of 50 mM HEPES (pH 7.0) was reduced with 0.5 mM dithiothreitol (DTT). The partially reduced HSA was supplied to a Hi Trap Capto Q column (GE Healthcare Biosciences) equilibrated with 50 mM HEPES buffer (pH 7.0), washed with the same buffer, and eluted with buffer containing 1 M NaCl. The eluted HSA (50 μM) was reacted with 250 μM dibenzocyclooctyne (DBCO)-PEG4-Maleimide (Click Chemistry Tools) and incubated for 2 hours at a room temperature. The DBCO-modified HSA formed (7 μM) was subsequently reacted with a 20-fold excess of DM1-SMCC (MedChemExpress) for 12 hours at 37°C.

After the reaction, the sample was supplied to a Hi Trap Capto Q column (GE Healthcare Biosciences) equilibrated with 50 mM HEPES buffer (pH 7.0), washed with the same buffer, and eluted with buffer containing 1 M NaCl. The drug (DM-1) attachment efficiency to the HSA (DAR) was estimated to be 2.9 using matrix-associated laser desorption/ionization time-of-flight mass spectroscopy (autoflex speed TOF; Bruker Daltonics).

Finally, azide-modified Z33-monovalent-modified antibody (10 μM) and DM-1/DBCO-modified HSA (20 μM) were reacted at pH 7.0 for 12 hours at room temperature through the click chemistry. The generation of antibody-HSA conjugate was confirmed by sodium dodecyl sulfate–polyacrylamide gel electrophoresis (SDS-PAGE). The reaction mixture was subjected to a Z33 affinity peptide column to remove the excess HSA eluted in the excluded fraction. The bound HSA-monovalent modified antibody was eluted by 0.1 M acetic acid buffer (pH 4.0) and dialyzed in PBS for use.

### Cell lines and animals

We previously inserted dPDPN with an N-terminal PA tag (GVAMPGAEDDVV)^(19)^ and a C-terminal RAP tag (DMVNPGLEDRIE)^(20)^-MAP tag (GDGMVPPGIEDK)^(21)^ (PA-dPDPN-RAP-MAP) in a pCAG-Ble vector (FUJIFILM Wako Pure Chemical Corporation).^(11)^ CHO-K1 cells (American Type Culture Collection, Manassas, VA) were transfected with pCAG-Ble/PA-dPDPN-RAP-MAP using Gene Pulser Xcell electroporation system (Bio-Rad Laboratories, Inc., Berkeley, CA) for developing CHO/dPDPN.

CHO/dPDPN and CHO-K1 cells were cultured using RPMI-1640 medium (Nacalai Tesque, Inc., Kyoto, Japan) supplemented with 10% heat-inactivated fetal bovine serum (Thermo Fisher Scientific, Inc.). Antibiotics, such as 100 U/mL penicillin, 100 μg/mL streptomycin, and 25 μg/mL amphotericin B (Nacalai Tesque, Inc.) were added into the medium. Cells were cultivated at 37°C in a humidified atmosphere of 5% CO_2_ and 95% air.

### Flow cytometry

Using 0.25% trypsin/1 mM ethylenediaminetetraacetic acid (Nacalai Tesque, Inc.), CHO/dPDPN cells were harvested. Cells were treated with P38B (0.01–10 μg/mL), P38B-DM1 (0.01–10 μg/mL), or normal canine IgG (10 μg/mL; Jackson ImmunoResearch, Inc., PA) for 30 minutes at 4°C, followed by treatment with fluorescein isothiocyanate-conjugated anti-canine IgG (1:200; Thermo Fisher Scientific, Inc.). In all steps, the cells were washed with 1% bovine serum albumin in PBS. Fluorescence data were detected using the Cell Analyzer EC800 (Sony Corp., Tokyo, Japan). We repeated these experiments twice.

### Cytotoxicity assay

Cytotoxicity assay by cell viability assessment was conducted using Cell Cloning Kit-8 (CCK-8; Dojindo, Kumamoto, Japan). We prepared 96-well microplates by plating CHO/dPDPN and CHO-K1 at 6000 cells/200 μL/well. The plates were incubated for 24 hours at 37°C in the presence of 5% CO_2_, then serially diluted antibodies were added in a volume of 20 μL and cultured for 24–72 hours. After that, the culture medium was replaced with 100 μL of fresh medium with 10 μL CCK-8 solution. After a further 5- to 6-hour incubation in a CO_2_ incubator, the absorbance at 450 nm was measured.

### Antitumor activity

Female BALB/c nude mice (5 weeks old) were purchased from Charles River (Kanagawa, Japan) and used in experiments when they were 6 weeks old. All animal experiments were reviewed and approved by the Institutional Animal Care and Use Committee of Institute of Microbial Chemistry (Approval number: 2019-043) and were performed in accordance with relevant guidelines and regulations to minimize animal suffering.

CHO/dPDPN cells (0.3 mL at 1.33 × 10^8^/mL in RPMI) and 0.5 mL of BD Matrigel Matrix Growth Factor Reduced (BD Biosciences, San Jose, CA) were mixed, and 5 × 10^6^ cells in 100 μL were injected into the left flank of nude mice subcutaneously. One day after cell inoculation, 100 μg of antibodies (P38B-DM1, P38B, or normal canine IgG) in 100 μL of PBS were injected into the peritoneal cavity of each mouse. On days 8 and 14, additional 100 μg of antibodies were injected into each mouse. As previously described, the tumor diameter and tumor volume were determined.^(22)^ The mice were killed 17 days after cell implantation.

All data were expressed as mean ± standard error of mean. Statistical analysis was performed using the Tukey–Kramer test, with *p* < 0.05 considered statistically significant.

## Results

**FIG. 1.**
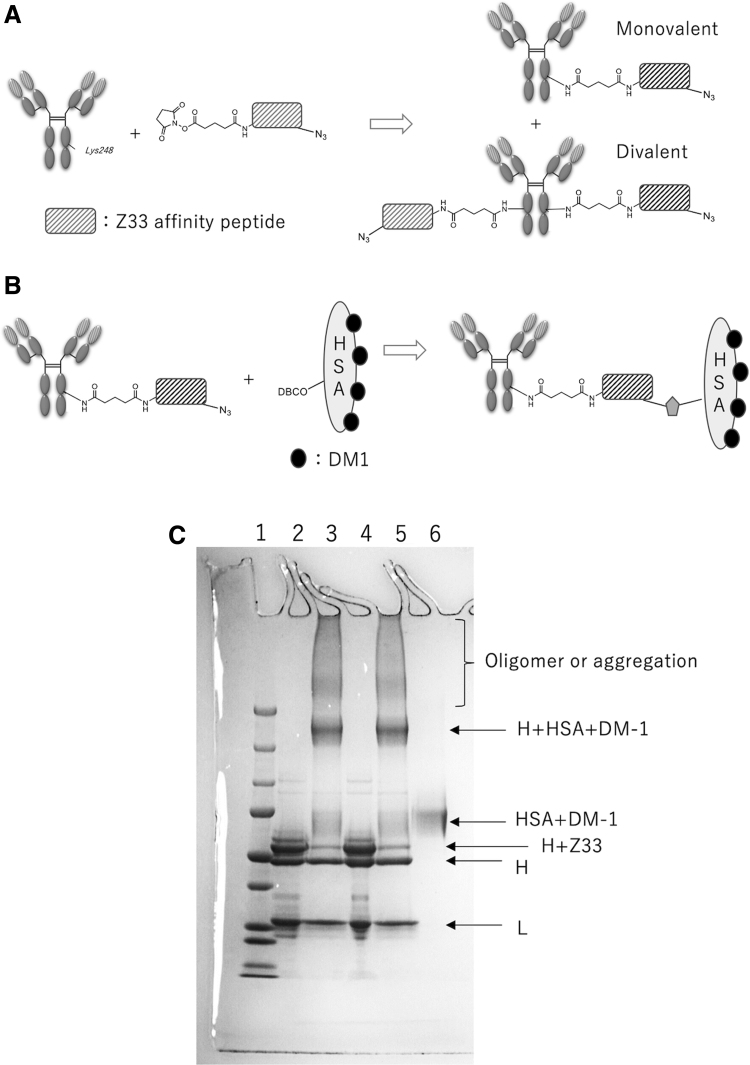
Preparation of ADC P38B-DM1 using CCAP method. **(A)** The reaction of antibody with CCAP peptide generated the monovalent and divalent antibodies owing to the two modified sites of CCAP peptide on Fc. To remove the unmodified and the divalent-modified antibodies, the reaction mixture was subjected to to affinity chromatography conjugated with Z33 affinity peptide. **(B)** The modification of HSA with DM-1 was performed. **(C)** Sodium dodecyl sulfate–polyacrylamide gel electrophoresis of conjugate products under reducing conditions. Lane 1: molecular weight marker; Lane 2: Z33-azide monovalent modified P38B; Lane 3: Z33-azide monovalent modified P38B + DBCO-HSA-DM-1; Lane 4: Z33-azide monovalent modified P38Bf (core fucose-deficient version of P38B); Lane 5: Z33-azide monovalent-modified P38Bf + DBCO-HSA-DM-1; Lane 6: DBCO-HSA-DM-1. H: heavy chain, L: light chain. DBCO, dibenzocyclooctyne; ADC, antibody–drug conjugate; CCAP, chemical conjugation by affinity peptide; HSA, human serum albumin.

In this study, we developed an ADC, P38B-DM1, using the mouse–canine chimeric anti-dPDPN antibody, P38B as the antibody, a peptide linker, and emtansine as the payload using the CCAP method as given in Figure 1A and B. SDS-PAGE of azide-modified Z33-monovalent modified antibody and its conjugate with DBCO-HSA-DM-1 (P38B-ADC) are given in Figure 1C.

**FIG. 2.**
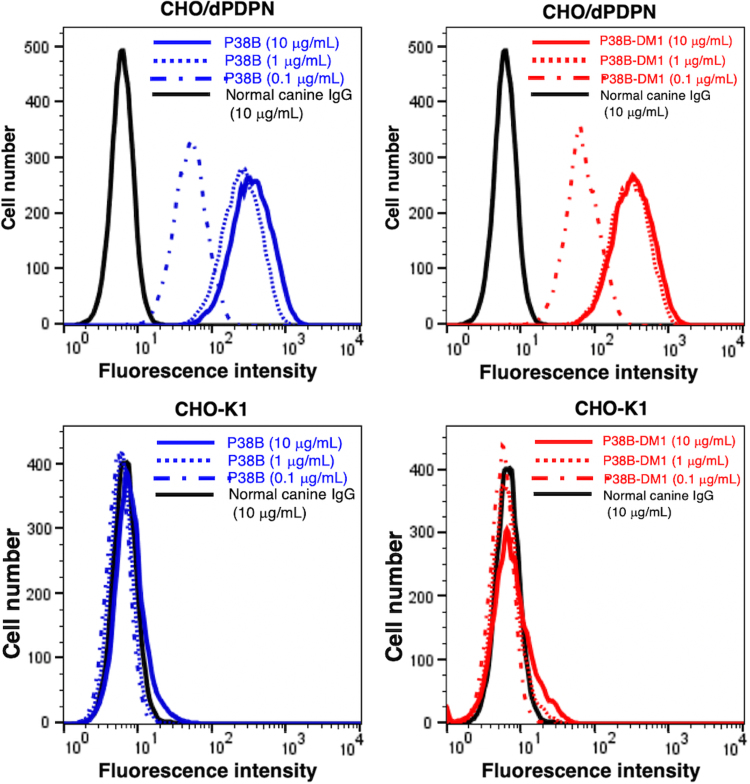
Flow cytometry. CHO/dPDPN cells were treated with P38B (blue line; 10 or 1 μg/mL), P38B-DM1 (red line; 10 or 1 μg/mL), and normal canine IgG (black line; 10 μg/mL), followed by treatment with fluorescein isothiocyanate-conjugated anti-canine IgG. Fluorescence data were collected using a cell analyzer. IgG, immunoglobulin G; CHO, Chinese hamster ovary; PDPN, dog podoplanin.

Antibodies are affinity proteins and their ability to bind the target molecule with high sensitivity and specificity is crucial to their function. With this in mind, we examined the characteristics of P38B and P38B-DM1. Flow cytometry results (Fig. 2) showed that both P38B and P38B-DM1 exhibited similar reactions with CHO/dPDPN cells in a dose-dependent manner. Furthermore, P38B-DM1 did not react with CHO-K1 (Fig. 2), indicating that it was sensitive and specific against dPDPN.

**FIG. 3.**
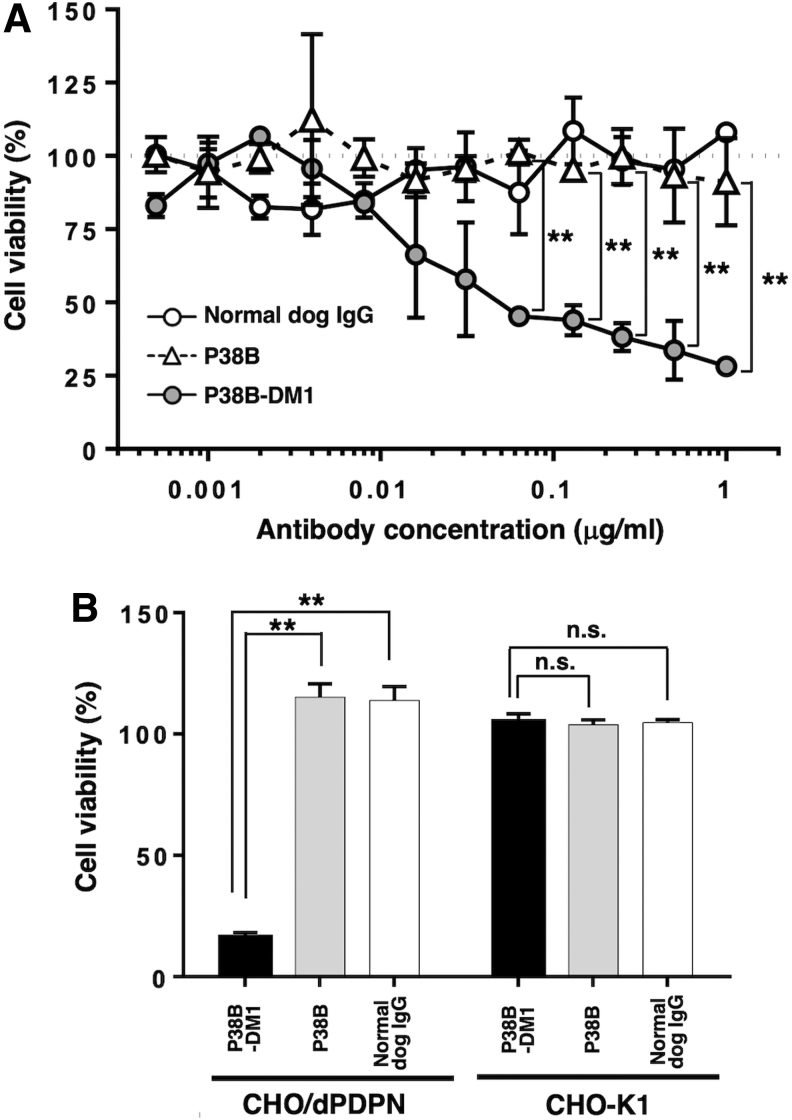
Cytotoxicity assay. **(A)** Cytotoxicity assay by cell viability assessment was conducted using CCK-8 We prepared 96-well microplates by plating CHO/dPDPN cells at 6000 cells/200 μL/well. The plates were incubated for 24 hours at 37°C in the presence of 5% CO_2_, then serially diluted antibodies were added in a volume of 20 μL and cultured for 24 hours. After that, the culture medium was replaced with 100 μL of fresh medium with 10 μL CCK-8 solution. After a further 5-hour incubation in a CO_2_ incubator, the absorbance at 450 nm was measured. The values are presented as mean ± SEM. Asterisks indicate statistical significance (**p < 0.01, Tukey–Kramer test). **(B)** Cytotoxicity assay by cell viability assessment was conducted using CCK-8 We prepared 96-well microplates by plating CHO/dPDPN and CHO-K1 cells at 6000 cells/200 μL/well. The plates were incubated for 24 hours at 37°C in the presence of 5% CO_2_, then serially diluted antibodies were added in a volume of 20 μL and cultured for 72 hours. After that, the culture medium was replaced with 100 μL of fresh medium with 10 μL CCK-8 solution. After a further 6-hour incubation in a CO_2_ incubator, the absorbance at 450 nm was measured. Values are presented as mean ± SEM. Asterisks indicate statistical significance (**p < 0.01, Tukey–Kramer test). n.s., not significant; CCK-8, Cell Cloning Kit-8; SEM, standard error of mean.

Next, we investigated the cytotoxicity of P38B and P38B-DM1 against CHO/dPDPN cells. P38B-DM1 demonstrated high cytotoxicity in a dose-dependent manner against CHO/dPDPN cells compared with P38B (*p* < 0.01, using Tukey–Kramer test in more than 0.0625 μg/mL) and normal canine IgG (*p* < 0.01, using Tukey–Kramer test in more than 0.0625 μg/mL) (Fig. 3A). In our previous study, P38B showed high ADCC in canine mononuclear cells, and showed CDC using rabbit complement in CHO/dPDPN.^(16)^ In this study, in contrast, the P38B-DM1 showed high cytotoxicity against CHO/dPDPN cells without mononuclear cell complement, indicating that it was internalized into CHO/dPDPN cells where DM1 exerted its cytotoxicity. We also performed additional experiments to confirm that P38B-DM1 did not show any toxicity against CHO-K1 cells. As given in Figure 3B, P38B-DM1 did not show any toxicity against CHO-K1 cells, whereas it killed CHO/dPDPN cells.

We further investigated antitumor activities of P38B-DM1 *in vivo*. CHO/dPDPN cells were subcutaneously implanted into the flanks of nude mice. P38B, P38B-DM1, and control (dog IgG) were injected three times (on days 1, 8 and 14 after cell injection) into the peritoneal cavity of the mice. Tumor formation was clearly observed in mice from the control, P38B-treated, and P38B-DM1-treated groups in CHO/dPDPN xenograft model.

**FIG. 4.**
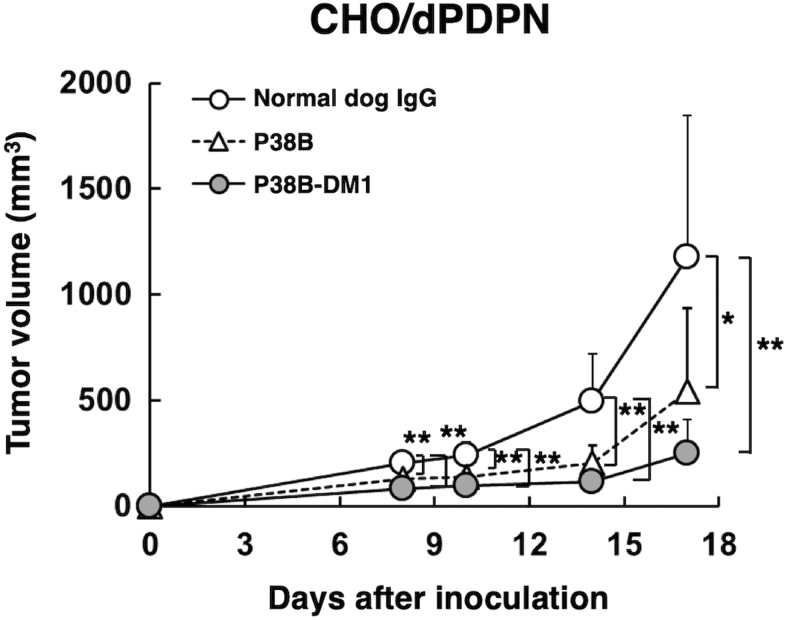
Antitumor activity of P38B and P38B-DM1 against CHO/dPDPN. Tumor volume of CHO/dPDPN xenografts. CHO/dPDPN cells were injected subcutaneously into female nude mice. The indicated antibodies (100 μg/day; 5 mg/kg) were administered intraperitoneally on days 1, 8, and 14 after cell inoculation. The tumor volume was measured at the indicated time points. The values are presented as mean ± SEM. Asterisks indicate statistical significance (*p < 0.05, **p < 0.01, Tukey–Kramer test).

We measured the tumor size in all the xenograft models and found that P38B-DM1 significantly reduced tumor development of the CHO/dPDPN xenograft compared with control (dog IgG) on days 8, 10, 14, and 17 (Fig. 4). However, the tumor volume in the P38B-DM1-treated groups was not significantly reduced compared with that in the P38B-treated groups because P38B also significantly reduced tumor development compared with control (dog IgG) on all days (Fig. 4). As reported in our previous study, P38B showed very high CDC activity against CHO/hPDPN cells,^(16)^ indicating that mouse complement of nude mice might capacitate CDC to P38B.

**FIG. 5.**
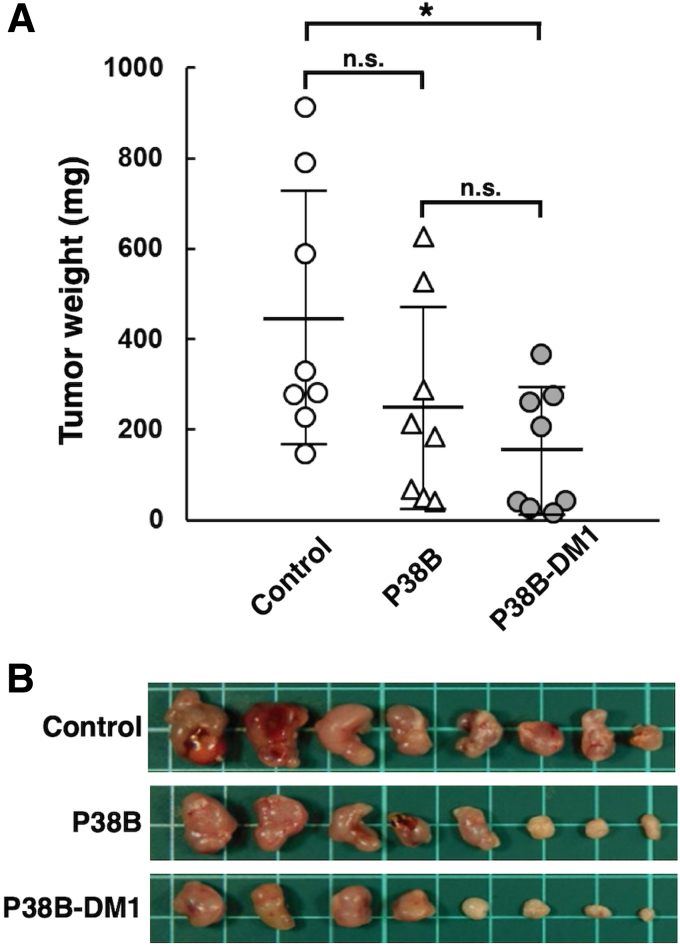
Evaluation of antitumor activity of P38B and P38B-DM1 against CHO/dPDPN. **(A)** Tumor weight of CHO/dPDPN xenografts (day 17). **(B)** Images of resected tumors of CHO/dPDPN xenografts. Values are presented as mean ± SEM. The asterisk indicates statistical significance (*p < 0.05, Tukey–Kramer test). n.s., not significant.

**FIG. 6.**
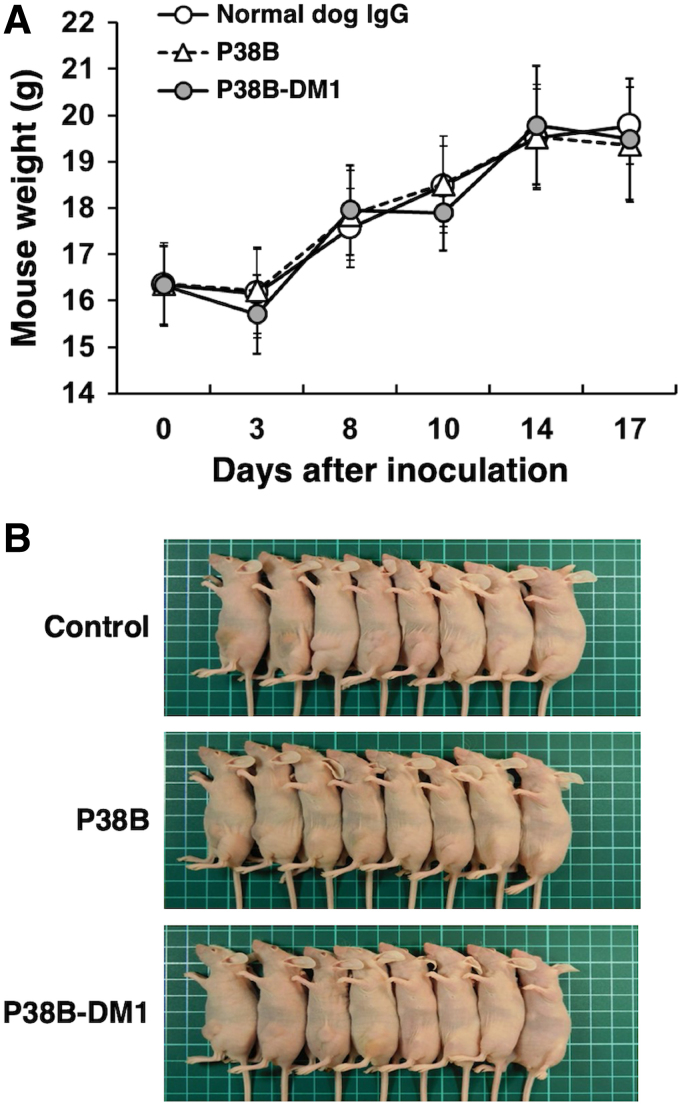
Evaluation of the safety of P38B and P38B-DM1 against CHO/dPDPN. **(A)** Body weight of CHO/dPDPN xenografts. **(B)** Images of CHO/dPDPN xenograft model mice on day 17.

We next measured the weight of resected tumors from all xenograft models on day 17. The tumor weight of mice in P38B-DM1-treated groups was significantly lower than that in the control (dog IgG) group in CHO/dPDPN xenograft models (Fig. 5A). In contrast, the tumor weight of mice in the P38B-treated groups was not significantly reduced compared with the control (Fig. 5A), indicating that P38B-DM1 exerted stronger antitumor activity than P38B. Images of the resected tumors of the CHO/dPDPN xenografts are given in Figure 5B. There were no differences in mouse body weight between the three groups (Fig. 6A), indicating that the P38B-DM1 was not toxic to the mice. The appearance of the mice on day 17 is given in Figure 6B. Because P38B-DM1 could not bind to Fc receptor of mouse mononuclear cells such as NK cells or macrophages, ADCC-mediated killing might not occur in the xenograft models. Although we could not exactly demonstrate the mechanism of anti-tumor activities by P38B-DM1 in this study, P38B-DM1 could be internalized into cells, and exerted cytotoxicity.

## Discussion

Cancer is a deadly disease that affects all eukaryotes. Almost all cancers found in humans are also observed in dogs, suggesting that the treatment approaches used in humans may also be effective in canine cancers. Antibody therapies have been shown to improve disease outcome in cancer patients and ADCs are thought to be one of the most potent next-generation antibody therapies.^(23)^

We previously produced P38B, a mouse–canine chimeric anti-dPDPN antibody of canine subclass B^(16)^ because canine subclass B possesses ADCC and CDC activities analogous to the human IgG_1_ subclass.^(24)^ We clearly demonstrated that P38B possesses ADCC and CDC against CHO/dPDPN cells.^(16)^ P38B reduced tumor development of CHO/dPDPN xenograft significantly compared with control dog IgG,^(17)^ indicating that P38B exerted antitumor activity against dPDPN-expressing tumors using ADCC and CDC activities. In those previous studies, canine mononuclear cells were also injected into xenograft models to induce ADCC activity of P38B. In contrast, antitumor activities of P38B were also observed in this study (Fig. 4), although canine mononuclear cells were not injected into the xenograft models, indicating that CDC of P38B alone could exert antitumor activity. However, the tumor weight of mice in the P38B-treated groups was not reduced significantly compared with the control (dog IgG) group in CHO/dPDPN xenograft models (Fig. 4A), suggesting that CDC of P38B alone did not exhibit sufficient antitumor activity.

In this study, an ADC, P38B-DM1 was generated by conjugating P38B and HSA-attached DM1 (Fig. 1). This ADC showed almost the same binding ability to dPDPN as unmodified P38B (Fig. 2), indicating that the HSA modification of the antibody did not affect antigen binding. Furthermore, its efficacy, as measured by its tumor regression activity, was confirmed by *in vitro* (Fig. 3) and *in vivo* (Figs. 4 and 5). P38B + DBCO-HSA-DM-1 conjugates will be intracellularly incorporated by endocytosis through the binding with dPDPN. The incorporated conjugate is degraded by proteases in lysosome to generate the DM1-attached Lys amino acid or oligopeptide, which can work as a killing agent through tubulin polymerization inhibition to induce apoptosis. The IC_50_ of P38B-DM1 was estimated to be 0.048 μg/mL in our preliminary experiment, which corresponds to 238 pM, assuming the molecular weight of P38B-DM1 is ∼210,000. This value is larger than the IC_50_ of T-DM1 (50 pM) against the HER2 + SK-BR3 breast cancer cell line despite the similar DAR for both (2.9 for P38B-DM1 and 3.5 for T-DM1). However, the difference in IC_50_ may be arise from a difference in the expression levels of the target antigens between the cell types. A detailed comparison in the near future is advised to elucidate the advantages of these ADCs.

In this study, we examined the effects of P38B, a mouse–canine chimeric anti-dPDPN antibody of subclass B and its ADC with emtansine (P38B-DM1) on CHO/dPDPN cells. Results indicate a significant increase in the cytotoxicity and antitumor activity of P38B-DM1 against CHO/dPDPN cells in comparison with P38B *in vitro* and *in vivo*, suggesting that P38B-DM1 is applicable for antibody therapy against canine cancers expressing dPDPN. Although the stability of our ADC has not been checked in serum or *in vivo*, the toxicity after administration *in vivo* has not been observed. This suggests that a rapid release of drug (DM1) from ADC did not occur after administration. In principle, we assume the action of mechanism of our ADC, as follows. ADC will be incorporated into the cells by endocytosis through the binding with PDPN. The incorporated ADC will be degraded in late endosome generated with fusion with lysosome into drug (DM1)-attached amino acid (Lys) or oligopeptides, which can work as a killing agent through the inhibition of tubulin polymerization. Further studies on antitumor activities against endogenous dPDPN-expressing tumors are necessary to obtain a more detailed understanding of antibody therapy against canine cancers.
